# How the Disruption of Mitochondrial Redox Signalling Contributes to Ageing

**DOI:** 10.3390/antiox12040831

**Published:** 2023-03-29

**Authors:** Beatriz Castejon-Vega, Mario D. Cordero, Alberto Sanz

**Affiliations:** 1 School of Molecular Biosciences, College of Medical, Veterinary and Life Sciences, University of Glasgow, Glasgow G12 8QQ, UK; 2 Department of Molecular Biology and Biochemical Engineering, Universidad Pablo de Olavide, 41013 Seville, Spain

**Keywords:** ageing, mitochondria, ROS, redox signalling

## Abstract

In the past, mitochondrial reactive oxygen species (mtROS) were considered a byproduct of cellular metabolism. Due to the capacity of mtROS to cause oxidative damage, they were proposed as the main drivers of ageing and age-related diseases. Today, we know that mtROS are cellular messengers instrumental in maintaining cellular homeostasis. As cellular messengers, they are produced in specific places at specific times, and the intensity and duration of the ROS signal determine the downstream effects of mitochondrial redox signalling. We do not know yet all the processes for which mtROS are important, but we have learnt that they are essential in decisions that affect cellular differentiation, proliferation and survival. On top of causing damage due to their capacity to oxidize cellular components, mtROS contribute to the onset of degenerative diseases when redox signalling becomes dysregulated. Here, we review the best-characterized signalling pathways in which mtROS participate and those pathological processes in which they are involved. We focus on how mtROS signalling is altered during ageing and discuss whether the accumulation of damaged mitochondria without signalling capacity is a cause or a consequence of ageing.

## 1. Introduction

**Figure 1:**
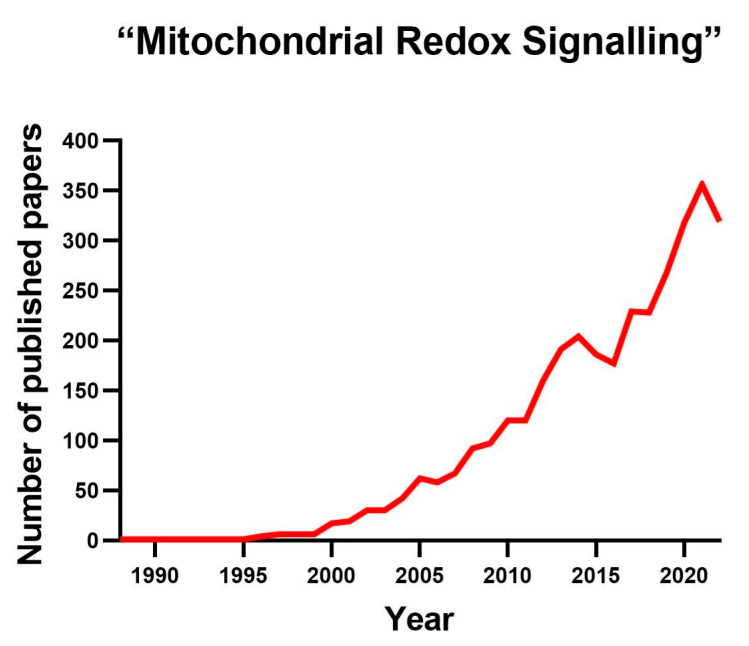
Number of published papers containing the expression “Mitochondrial Redox Signalling” in their titles or abstracts, as extracted from the Scopus database. The y-axis indicates the number of publications per year, and the x-axis shows the years included in the search. The data provide insights into the growing popularity of redox biology within the scientific community.

Reactive oxygen species (ROS) is an umbrella concept that includes multiple molecules resulting from the incomplete reduction of oxygen to water. The former occurs when oxygen is reduced with three, two or one electron [1]. ROS can attack other molecules, modifying their structure; by doing so, they change the way the modified molecule behaves [1]. While some ROS, such as superoxide, are free radicals, others, such as hydrogen peroxide, are not. Initially, ROS were considered toxic agents responsible for ageing and age-related diseases. This concept originated within the free radical theory of ageing framework, which presents ROS as simple byproducts of cellular metabolism [2,3]. However, we know that ROS are also messengers controlling critical cellular functions, and the interest in studying them has exponentially increased in recent years (Figure 1). ROS can be produced both externally and internally. Ultraviolet light, ionizing radiation and smoking are external sources of ROS, whereas internally, ROS are generated during inflammation, protein folding, fatty acid oxidation or mitochondrial respiration. Although ROS can be generated in various locations within a cell, this review will concentrate solely on ROS produced within the mitochondria, known as mitochondrial ROS (mtROS). We refer the reader to other excellent papers in which extra-mitochondrially produced ROS are exhaustively discussed [4,5,6,7].

Mitochondria are double-membrane organelles with their own DNA [8]. Within mitochondria, the generation of ROS is mainly associated with oxidative phosphorylation (OXPHOS). The mitochondrial electron transport chain (ETC) is canonically composed of respiratory complexes I-IV. As electrons move through the ETC, energy is released to move protons into the intermembrane space, creating an electrochemical gradient. Complex V then uses this gradient to convert ADP to ATP through a process called oxidative phosphorylation (OXPHOS) [9]. Mitochondrial ATP generation is essential for cell survival; however, ATP production is just one of the many functions performed by mitochondria. Most mitochondrial functions require a functional ETC to be completed. Calcium homeostasis and synthesis of iron–sulphur clusters and pyrimidines are just a couple of examples of the essential tasks performed by mitochondria coupled with OXPHOS [10]. For the ETC to work correctly, it is necessary to transfer electrons to the final electron acceptor, oxygen. When oxygen receives four electrons, it is completely reduced to water; however, when it receives one, two or three electrons, ROS are formed [1]. Superoxide results from the reduction of oxygen with one electron, the reduction of hydrogen peroxide with two electrons and the reduction of a hydroxyl radical with three electrons. In addition, the former ROS can further react with other molecules to produce more damaging chemicals, such as peroxynitrite [11].

Molecules that neutralize ROS are called antioxidants. However, the existence of oxidized proteins, lipids and DNA shows that antioxidants are not 100% efficient [12]. Because ROS are not only byproducts of metabolism, antioxidants are not only detoxifiers; they are also information transducers, allowing, for example, for the transformation of a superoxide signal into a hydrogen peroxide signal [13]. There are both enzymatic and non-enzymatic antioxidants [14]. Enzymatic antioxidants are specific against superoxide (superoxide dismutases) or peroxides (catalases, peroxiredoxins or glutathione peroxidases). In contrast, non-enzymatic antioxidants such as vitamin C or E are non-specific and intercept more damaging ROS such as hydroxyl radicals. The discovery of the enzyme superoxide dismutase confirmed the generation of ROS within the cell [15]. Superoxide dismutases dismutate superoxide to hydrogen peroxide. There are three different enzymatic systems dedicated to detoxifying hydrogen peroxide: (1) glutathione peroxidases, (2) peroxiredoxins and (3) catalases. Catalases decompose hydrogen peroxide to oxygen and water. Peroxide oxidizes glutathione peroxidases and peroxiredoxins, which are then reduced by thioredoxin and glutathione. Finally, NADPH, which is produced mainly by the pentose phosphate pathway, is the final electron donor for thioredoxin and glutathione regeneration as the last electron donor. The former shows that maintaining redox homeostasis requires constant metabolic adjustments [16]. 

One of the most important questions in redox biology is how ROS signals are transmitted. The most popular answer is that redox information is transmitted through the reversible oxidation of specific cysteine residues [17]. However, oxidative modifications of other amino acids, such as methionine, are also important [18]. Peroxiredoxins intercept most of the hydrogen peroxide produced by cells [19]. When catalase detoxifies hydrogen peroxide, the information is lost. However, oxidized peroxiredoxins can convey the information carried by hydrogen peroxide. Four models have been proposed to explain how ROS act as cellular messengers, as reviewed in [20]. In two of these models, peroxiredoxins are instrumental. The first model proposes direct interaction between ROS and the target protein, avoiding antioxidant interception. The second suggests binding the target protein to the generator of ROS. The third model, called the floodgate model, proposes the hyper-oxidation of peroxiredoxins as the mechanism required for transmission of the redox signal. Finally, the relay model states that (oxidized) peroxiredoxins directly oxidize the target protein, modifying its function. The last two models are the most supported by experimental evidence [21,22]. An example of the relay model occurs in *Caenorhabditis elegans* in response to metformin feeding [23]. Inhibition of CI by metformin triggers ROS production, causing the oxidation of PRDX-2 and subsequent activation (by the latest) of an MAPK cascade that prolongs the worms’ lifespan.

## 2. Four Parameters to Describe the Functions of mtROS

**Figure 2:**
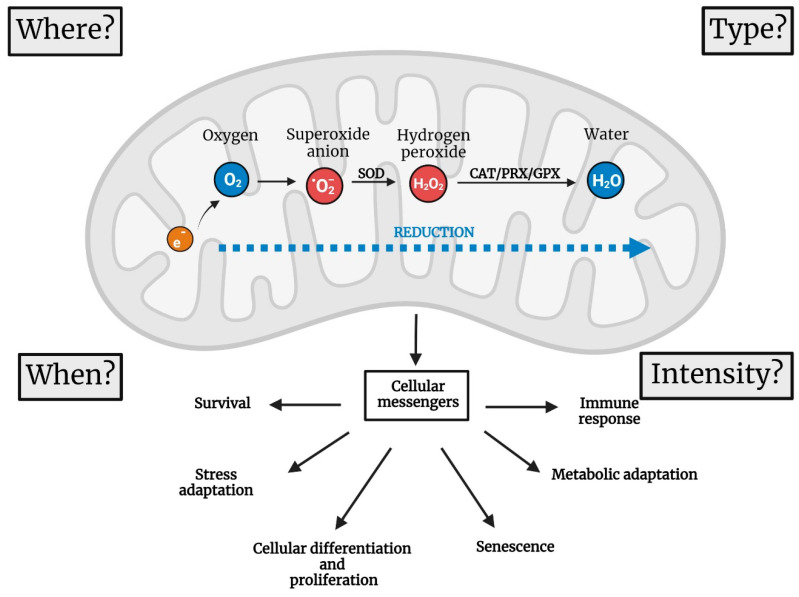
Mitochondrial reactive oxygen species (ROS) are important cellular messengers, but understanding their role in cellular physiology requires consideration of four questions: (1) Where is the signal produced?; (2) When is the signal produced?; (3) What type of signal is produced?; and (4) What is the intensity of the signal? These four characteristics of an mtROS signal determine the downstream effects it can induce. Antioxidants act as essential regulators of ROS signalling by either transforming one signal into another, regulating the intensity of the signal or turning off the signal. The figure depicts some examples of the processes controlled by mtROS signalling. For more information, please refer to the main text.

To understand the physiological role of mtROS, it is imperative to recognize them as signalling molecules that are generated to transmit information and exert an effect. Therefore, they can cause damage by triggering oxidative stress or defects in signalling processes. Since ROS can function as cellular messengers, it is important to consider four aspects when studying their roles (Figure 2): (1) where ROS are produced, (2) when ROS are generated, (3) the type of ROS and (4) the amount of ROS or intensity of the ROS signal. Studying ROS as signalling agents requires an appropriate experimental setup. Here, we do not pretend to revise the different ways of measuring mtROS or provide advice on performing such measurements. Therefore, we refer the reader to other excellent reviews and technical papers [1,24]. Nevertheless, we will briefly discuss how ROS are measured to provide the appropriate context for the rest of this review. For historical reasons, most information about how mitochondria work and produce ROS comes from studies using isolated mitochondria. Isolated mitochondria measurements provide a high level of resolution, allowing us to identify which respiratory complex generates the ROS. However, they lack physiological relevance (Sanz 2016). These experiments are also performed in the context of supraphysiological oxygen levels, causing overoxidation of mitochondrial components. High resolution is achieved using isolated mitochondria via the combination of specific substrates and inhibitors of different ETC complexes [25]. Working with intact cells increases the physiological relevance of mtROS experiments. However, the problem with supraphysiological oxygen levels remains the same unless it is adequately addressed within a normoxia chamber [26]. Furthermore, in vitro cell measurements do not provide sufficient resolution to identify the generator of ROS per se. Resolution is increased by using inhibitors similar to those employed in isolated mitochondria or a genetic approach to silence the ROS generator. For obvious reasons, the most informative measurements are those performed in vivo using animal models such as worms [27], flies [28] or mice [29]. Finally, mtROS measurements must be validated using different methods. Validation with antioxidants serves a dual purpose. First, it confirms the type of ROS being assessed. For example, superoxide dismutase overexpression reduces superoxide, while catalase diminishes hydrogen peroxide levels [30]. The physiological importance of the signal is confirmed if the analysed phenotype is modified accordingly due to the change in antioxidant levels [31].

**Figure 3:**
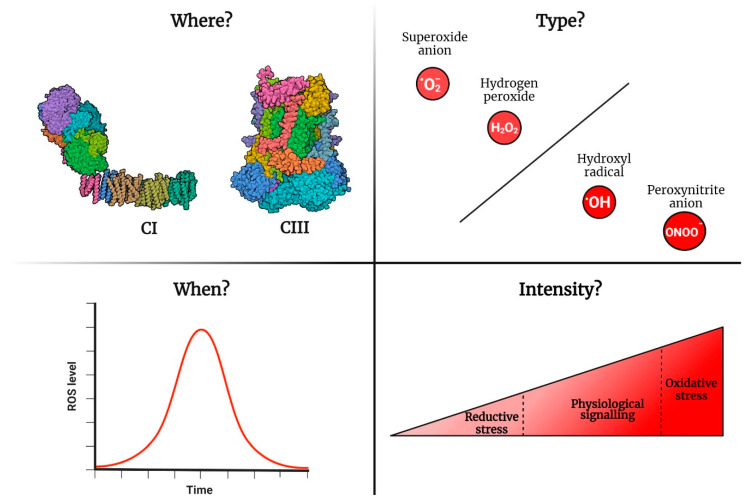
The four aspects of an mtROS signal that impact its downstream effects. “Where” indicates that ROS produced by complexes I and III participate in various physiological and pathological processes. “When” refers to the temporal dynamics of mtROS signalling and highlights the importance of understanding how the signal is initiated and terminated using kinetic measurements rather than a single-point measurement. “Type” explains that mtROS signalling is mainly mediated by low reactive species, such as superoxide and hydrogen peroxide, while highly reactive species, such as hydroxyl radical, are responsible for oxidative damage. Finally, “Intensity” emphasizes that the intensity of mtROS signals needs to be within an appropriate physiological range to avoid disrupting cellular homeostasis by triggering oxidative or reductive stress. The figure illustrates the interplay between these four aspects of mtROS signalling and their downstream effects.

As mentioned before, the characterization of ROS signalling involves determining the intensity of the signal, in addition to when and where the signal is generated and the type of signal (superoxide, hydrogen peroxide, etc.). In the following sections, we will discuss examples of the importance of specific spatiotemporal ROS signalling in physiological and pathological situations and argue why ROS signalling becomes problematic during ageing (Figure 3).

### 2.1. Where Are mtROS Produced?

For many years, mitochondria were considered the main generator of ROS [32]. The former is supported by mitochondria consuming most of the oxygen cells use. However, a few recent studies have questioned the former [5,33,34]. According to these studies, mitochondria account for about 50% of the hydrogen peroxide that is released from a cell. The remaining 50% is produced by enzymes called NADPH oxidases (NOX). However, since the detection system was located outside the cell, the former experiments did not account for the hydrogen peroxide (or superoxide) intercepted by intracellular antioxidants. Accordingly, studies in yeast using genetically encoded hydrogen peroxide reporters (that detect hydrogen peroxide generated within the cell) showed higher levels of hydrogen peroxide in the mitochondria than in the cytosol [35,36]. Regrettably, these reports did not quantify the levels of hydrogen peroxide in other significant generators of ROS, such as peroxisomes, the endoplasmic reticulum and lysosomes. Therefore, it is not possible to conclude that mitochondria are the main ROS generator. In the future, we will have to quantify ROS generated simultaneously at different locations to determine their relative importance depending on the physiological conditions. 

Even if mitochondria are not the main generators of ROS within the cell, mtROS play a significant role in redox signalling [37]. Up to 11 ROS generators have been found in isolated mitochondria [34]. However, most do not generate significant amounts of ROS in vivo. Under physiological conditions, complex I (CI) and complex III (CIII) are the main physiological ROS producers [31]. Complex II (CII) produces ROS only in pathological situations such as cancer or neurodegenerative diseases [38]. CI generates ROS in both the forward and reverse directions [39]. In the forward direction, ROS production depends on the redox state of the NAD(H) pool, whereas in the reverse direction, it depends on the redox state of the coenzyme Q (CoQ) pool and the proton motive force (pmf) [31,40]. The generation of CIII ROS relies on the entry of electrons through different mitochondrial dehydrogenases (e.g., CI and CII), the redox state of CoQ and the pmf [41,42]. Supporting a leading role of CI/CIII in ROS generation, specific suppressors of superoxide production in CI (S1QELs) and CIII (S3QELs) have been recently developed [43,44]. S1QELs and S3QELs do not alter electron flow or oxygen consumption but prevent the reduction of oxygen to superoxide by leaked electrons from the ETC. S1/3QELs represent convenient research tools with potential as therapeutic agents. Accordingly, they have demonstrated efficacy in protecting against excessive ROS levels in the heart, liver and gut [43,45]. 

When we discuss redox signalling, an additional layer of complexity is that mitochondria produce mtROS in different ways depending on the cell type. For example, mitochondria from glial cells generate more mtROS than neuronal mitochondria [46]. The former is caused by higher levels of free CI in glial cells. Furthermore, different types of mitochondria within a cell also generate ROS differently. For example, in rat muscle, subsarcolemmal mitochondria generate more hydrogen peroxide than interfibrillar mitochondria [47]. The variety in terms of how different cells and mitochondria produce ROS needs further study, since specific physiological and pathological processes are linked explicitly with mtROS produced by CI or CIII [31]. Furthermore, cellular differentiation is a process in which mtROS play a leading role. Accordingly, mtROS are essential to correctly differentiate the haematopoietic cells of flies [48]. Interestingly, some cell types require CI or CIII ROS to differentiate appropriately. CI ROS are specifically needed to transform myoblasts into myotubes [49]. During the differentiation process, myoblasts start to produce ROS via reverse electron transport (RET), and suppression of the former by rotenone stops the differentiation process. Adipocytes also use an mtROS signal to become fully differentiated [50]. However, in the case of adipocytes, the signal is produced by CIII, not CI. Accordingly, the knockdown of the CIII Rieske Fe-S subunit prevents ROS generation and stops the specialization of adipocytes. 

Different aspects of the immune response seem to be controlled explicitly by ROS generated in CI or CIII [51]. For example, proinflammatory reprogramming of macrophages in response to infection requires CI ROS [52], while activation of T cells is reported to be mediated by CIII ROS [53]. As experiments implicating CI or CIII in ROS generation are typically conducted by different research teams using various experimental setups, we cannot rule out the possibility that ROS produced by other complexes may also be necessary (or sufficient). To determine the veracity of these findings, it is advisable to repeat these experiments using multiple approaches to inhibit ROS production by both CI and CIII under identical experimental conditions.

### 2.2. When Are ROS Produced?

As with all signals, ROS signals must be turned on and off. Due to practical constraints, ROS measurements are generally presented as static, single-time-point values rather than dynamic processes that evolve over time. Nonetheless, the literature offers instances of mtROS signalling whereby an mtROS signal is triggered by specific stimuli and is concluded when the stimulus subsides. The most conspicuous example is the adaptive response to cellular hypoxia. In response to hypoxia, CIII produces ROS instrumental in stabilizing HIF1a [54]. Chemical (with myxothiazol) or genetic suppression (using cytochrome b mutants or RNA interference against the Rieske protein) of mtROS prevents the adaptation response to hypoxia [55,56]. Once the initial hypoxic response (i.e., stabilization of HIF1a) is completed, the CIII ROS signal is turned off [57,58]. Another example of mediation by CI ROS is how the specialized cells of the carotid body detect and react to low levels of oxygen in the blood [59,60]. These chemoreceptor cells have a specific mitochondrial metabolism characterized by high succinate levels. The former favours ROS generation via RET in response to low oxygen concentration. ROS-RET activation leads to elevated respiration, which helps to restore normal oxygen levels in the bloodstream. Once this has been achieved, the signal ceases. Similarly, macrophage activation in response to infection uses a similar ROS-RET mechanism that is also terminated when the damage signals disappear from the tissue [52].

By no means does the signal’s duration determine the importance of the downstream processes triggered by it. For example, fly brain mitochondria produce an ROS-RET signal in response to thermal stress [61]. Although the signal only persists for a few hours, it is vital for stress adaptation. If the signal is disrupted, fly survival under stress is significantly reduced. Similarly, ROS levels during the early stages of worm development determine the duration of the adult lifespan [62]. Worms with higher levels of ROS during L2 are long-lived, and experimental manipulations that elevate ROS in this period extend their lifespan. This phenomenon is conserved in fruit flies, where exposure to oxidants during development increases longevity [63]. On the other hand, extending ROS signalling for too long has harmful consequences. An excellent example of the former is tissue repair, which is controlled by TGF-beta signalling. The former stimulates CIII ROS production, which is instrumental in completing the healing process in the lungs [64]. Preventing the generation of ROS impairs the repair activity. However, excessive or sustained CIII ROS signalling can result in fibrosis, highlighting the need to regulate ROS signalling with appropriate intensity and duration. Another example of the former is pulmonary vasoconstriction. Low oxygen levels trigger CIII ROS signalling in the lungs [65,66]. ROS signalling plays a crucial role in triggering the vasoconstriction response, which preserves the integrity of the lungs. Therefore, insufficient or excessive ROS signalling can compromise lung integrity and endanger the survival of the individual [67]. 

Finally, how is the ROS signal turned on and off? Although this is a question of crucial importance, it is one of the most understudied aspects of redox signalling. ROS-RET signalling is started by increasing the entry of electrons to the ETC. This is achieved by enhancing access via single or multiple entry points. An example of single-point entry is ischemia–reperfusion. During reperfusion, CII oxidizes the succinate accumulated during ischemia, triggering ROS-RET [68]. However, ROS-RET is activated by electrons introduced via CI, CII and other mitochondrial dehydrogenases (not part of the canonical ETC) in response to thermal stress in fly brain mitochondria [61]. Recently, it was reported that oocytes have negligible amounts of CI [69]. This was interpreted as a mechanism to prevent the accumulation of oxidative damage in cells that are extremely important for reproduction. However, preventing CI assembly could be a way to keep signalling inactive until it is required, i.e., after fertilization. Accordingly, the assembly of CI upon fertilization triggers mtROS signalling, which is instrumental in correctly initiating the developmental program [70]. 

An interesting new mechanism has been described to explain how CIII ROS signalling initiates the hypoxia response [58,71]. Hypoxia deactivates CI, leading to acidification of the mitochondrial matrix and the exchange of mitochondrial Ca^2+^ with cytosolic Na^+^. The increased Na^+^ levels within the mitochondrial matrix limit the mobility of CoQ, resulting in a longer semiquinone lifetime within CIII and the subsequent generation of superoxide [58]. Investigating whether this mechanism is replicated in other situations in which CIII-derived ROS play a crucial role is imperative. There are several ways to turn off mtROS signalling. Unfortunately, there are not many studies addressing this vital issue. One possibility is to reduce the entry of electrons into the ETC. This has been shown to be effective in cancelling ROS-RET and CIII ROS signalling [61,68,72]. Another alternative is to increase the levels of antioxidants, which eliminates the benefits conferred by increased mtROS levels during exercise in humans [73]. 

## 3. Pathological Role of mtROS

### 3.1. The Two Ways by Which ROS Cause Damage

There are two ways ROS cause cellular distress: dysregulation in redox signalling and oxidative damage. Aberrant redox signalling occurs when signals are not produced or are generated at the incorrect time/place or with the wrong intensity [74]. We presented a few examples of the former above. Damage is provoked by too much signalling or a lack thereof. Ablation of mitochondrial redox signalling in mouse astrocytes causes metabolic and behavioural alterations [29]. On the other hand, overexpression of the ATPase inhibitory factor 1 (IF1) in mouse neurons increases mtROS generation, boosting the learning skills of mice [75]. Conversely, knocking out IF1 impairs memorization and performance in different cognitive tests. The other way ROS cause harm is through oxidative damage. High concentrations of ROS trigger oxidative stress [76]. This happens when the concentration of oxidants exceeds that of antioxidants, leading to oxidative damage [11]. For instance, animals fed oxidants such as hydrogen peroxide or paraquat exemplify the former scenario and die as a result [77]. Likewise, in mice, SOD2 knockout is lethal during embryonic development [78]. Additionally, ischemia–reperfusion provides a more physiological example of oxidative stress culminating in cell death [79]. As we have seen, ROS-RET triggered by succinate accumulation during ischemia causes oxidative damage, subsequently triggering tissue damage [68]. Accordingly, preventing RET attenuates oxidative stress and preserves the function of the affected tissue [43,68,80,81]. Some cancers are caused by mutations in genes encoding CII subunits that increase mtROS levels [82,83]. Recent studies have revealed that ROS-RET is upregulated in cancer stem cells with aberrant NOTCH signalling, a critical factor in the growth of tumour cells in mouse and fly brain models. [84]. Tumour necrosis factor (TNF) is critical to protect cells against mycobacteria [85]. However, excessive TNF signalling triggers ROS-RET, which causes necrosis in macrophages infected by mycobacteria [86]. Accordingly, metformin inhibits both ROS-RET and macrophage death. These findings open the possibility of using metformin in combination with antituberculosis drugs to prevent the harmful effects of the latter. 

Several diseases are associated with excessive CIII ROS. As previously noted, unchecked CIII ROS generation can lead to lung fibrosis [64]. Similarly, pollution causes thrombosis in the lungs by triggering CIII ROS [72,87]. Like CI ROS, CIII ROS play a crucial role in the proliferation and metastasis of specific tumour types, such as those regulated by KRAS signalling [88,89]. Again, it remains unclear whether CI/CIII ROS are responsible for the proliferation and metastasis of specific tumour cells or if the divergent outcomes are attributable to methodological differences in the execution of the experiments. Finally, some unidentified in vivo ROS generators may be crucial for other afflictions, since at least 11 sites produce superoxide in isolated mitochondria [90]. 

### 3.2. The Role of mtROS in Ageing

Damage caused by endogenously produced mitochondrial free radicals was initially proposed as the cause of ageing by Denham Harman [2,3]. In his famous “Mitochondrial Free Radical Theory of Ageing” (MFRTA), Harman proposed that mtROS produced as byproducts of metabolism cause the accumulation of oxidative damage responsible for ageing and age-related diseases. Over the years, the MFRTA has been supported by two types of evidence [91]: (1) observational studies describing the age-related accumulation of oxidative damage [92,93] and (2) correlative studies showing that mitochondria from long-lived animals produce fewer ROS [94,95]. While the theory was highly prevalent at the turn of the 21st century, it has since experienced a significant decline in popularity and is now considerably less popular than it once was [96,97]. The demise of MFRTA began when its forecasts were subjected to experimental verification. The first prediction states that increasing levels of antioxidants will extend lifespan and that, conversely, decreasing their concentration will shorten survival. Supplementing non-enzymatic antioxidants fails to increase longevity in most animal models [94]. Similarly, overexpression of enzymatic antioxidants reduces oxidative stress but does not extend the lifespan of mice [98,99]. An important exception is the targeted expression of catalase to mitochondria, significantly improving the median and maximum survival of mice [100]. However, the same strategy in fruit flies shortens lifespan [101] and causes deleterious alterations in mouse behaviour [29]. Unexpectedly, genetic depletion of most enzymatic antioxidants elevates oxidative damage without impacting mouse survival [102,103,104]. Nevertheless, there are some exceptions, such as knocking out SOD1 in mice or knocking down peroxiredoxins 3 or 5 in fruit flies [105,106]. However, these three enzymes are instrumental in redox signalling, and therefore, it is impossible to conclude whether the animals die because of the accumulation of oxidative damage, interruption in redox signalling or both. 

The second prediction of MFRTA argues that decreasing mtROS levels must extend lifespan and that increasing mtROS generation must shorten survival. However, both in worms and flies, we find examples of experiments disproving both predictions. Ectopic expression of the alternative oxidase (AOX) from *Ciona intestinalis* in *Drosophila* mitochondria reduces mtROS but does not extend lifespan [107,108]. In *Caenorhabditis elegans*, it is possible to increase mtROS production by using inhibitors of the ETC or knocking down subunits of the respiratory complexes. Both types of interruptions in electron flow increase longevity [109]. To the best of our knowledge, ETC inhibitors have not been reported to extend the lifespan of fruit flies, although inhibition of CV does (see below). Surprisingly, feeding rotenone to killifish extends longevity by 15% [110]. In flies, the knockdown of CI, CIII, CIV and CV extends survival [111]. The most consistent results are obtained by knocking down CI subunits, where lifespan is prolonged by an mtROS-dependant mechanism [112]. However, a recent report showed a reduction in mtROS associated with CI depletion in *Drosophila* flight muscle and extended lifespan [113]. Extending the lifespan of Drosophila is achieved by expressing the internal alternative NADH dehydrogenase 1 (NDI1) from Saccharomyces cerevisiae [114,115]. NDI1 promotes longevity, stimulating the generation of mtROS via RET. Similarly, several reports show that chemical or genetic inhibition of ATP synthesis by blocking CV increases mtROS levels and the survival of fruit flies and worms [116,117,118,119]. In summary, all the former results do not support the role of mtROS limiting lifespan per se. Conversely, they indicate that adequate mitochondrial redox signalling is essential for a healthy lifespan. As we will see next, mitochondrial redox signalling becomes a problem for aged individuals. 

### 3.3. Accumulation of Damaged Mitochondria during Ageing Causes an Interruption in Mitochondrial Redox Signalling

We have seen that mitochondrial redox signalling is a sophisticated communication system that becomes a source of damage during ageing. Controlled and regulated occurrence of ROS-RET and CIII ROS production requires specific conditions [40]. For example, ROS-RET requires the appropriate levels of reduction of the CoQ pool and a sufficiently high pmf to provide the necessary energy for RET [120]. In the ageing process, a significant concern arises due to the difficulty experience by old mitochondria in maintaining a high mitochondrial membrane potential [121]. Thus, the age-related decline in the NAD+-to-NADH ratio can potentially modify how CI generates ROS [122]. Furthermore, the depletion of NAD+ has the potential to impact the activity of SOD2, as it necessitates deacetylation by SIRT3 for activation, and SIRT3, in turn, is reliant on NAD+ [123]. This may also impede the transformation of superoxide signals into more stable hydrogen peroxide signals, thereby modifying the cellular signalling process. Initiating the CIII signalling process requires an influx of electrons, while a proper efflux is necessary to conclude the process [31]. Therefore, any process that alters the entry or exit of electrons, such as those associated with ageing, as described below, also modifies CIII redox signalling. In conclusion, it is unsurprising that alterations in the mitochondrial electron flow caused by the disruption in the OXPHOS machinery profoundly affect redox signalling [48,124]. Accordingly, one of the hallmarks of ageing is the accumulation of dysfunctional mitochondria that generate fewer ATP and higher levels of mtROS [125]. 

Reduced mitochondrial oxygen consumption is a common characteristic of ageing found in multiple animal species [28,126,127,128]. The referred decrease is related to problems with both the entry (due to problems with CI) and the exit of electrons (caused by issues with CIV) [28,129,130,131,132]. Age-related reduction in the levels of CI has been reported in the human cortex [133], naked mole rat skeletal muscle [134]) and fruit fly mitochondria [135]. However, other studies inform of a preferential decrease in CIV activity correlated with high levels of mtROS in flies and mouse adipocytes [131,136]. Reducing the entry of electrons can impede the generation of ROS signals [30], while blocking their exit results in the cessation of ROS signalling due to the ongoing production of uncontrolled ROS [61]. Interestingly, the opposite also occurs, i.e., increased mitochondrial oxygen consumption that disrupts mitochondrial redox signalling during ageing. The former happens during cellular senescence when an expansion in mitochondrial mass increases both cellular oxygen consumption and levels of ROS [137]. In senescence cells, mtROS reprogram the cell, causing DNA damage and releasing proinflammatory cytokines such as IL-6. Accordingly, strategies that reduce either the number of mitochondria or the amount of ROS decrease DNA damage and the senescence-associated secretory phenotype [138]. All the examples presented above indicate that age-related alterations in mitochondrial respiration disrupt physiological redox signalling. Notably, modifications in redox signalling can arise through different mechanisms, which can entail increments and decrements in oxygen consumption. A significant challenge for future research is to conduct tissue-specific analysis of mitochondrial function, investigate how these alterations affect redox signalling and explore the potential consequences for the cellular processes regulated by mtROS. 

**Figure 4:**
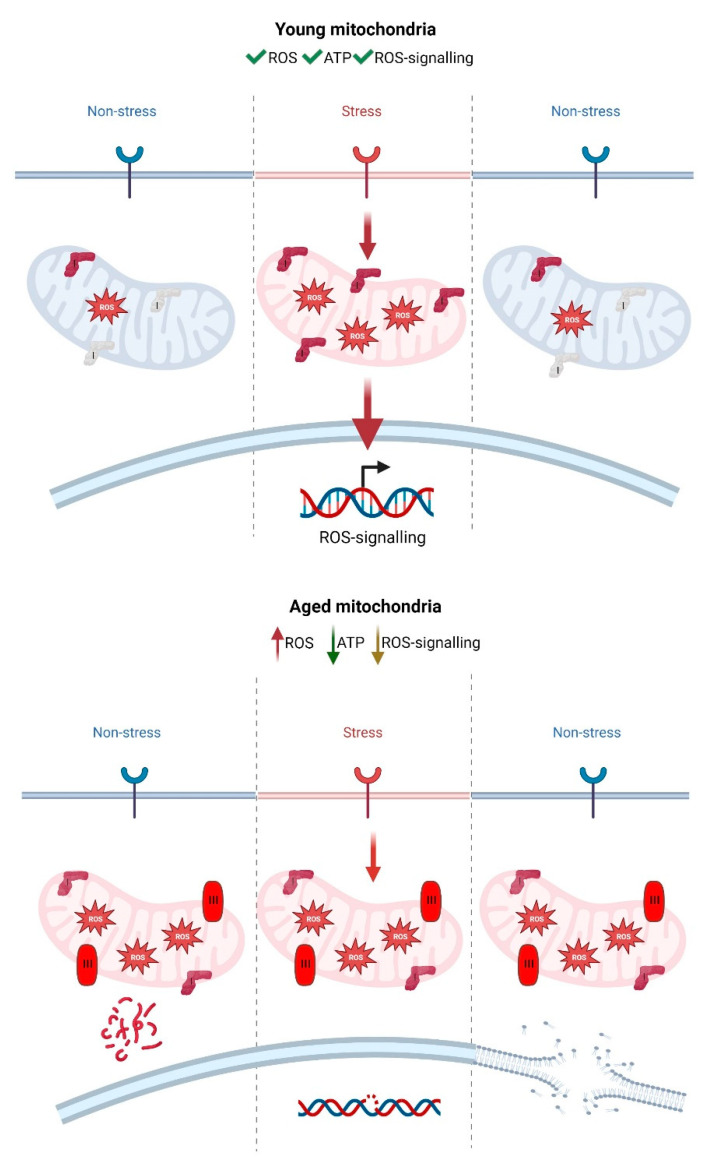
Mitochondrial redox signalling is altered during ageing, with young mitochondria producing ROS only in response to specific stimuli, thereby maintaining low total ROS levels. Conversely, ageing mitochondria continually produce high levels of ROS and are unresponsive to external stimuli, resulting in oxidative stress and damage. Antioxidants alone are not effective in increasing lifespan, as, while they can reduce oxidative damage, they cannot restore redox signalling. Effective antiageing therapies require not only a reduction in oxidative damage but also restoration of mitochondrial ROS (mtROS) signalling. The figure illustrates the contrasting differences between young and ageing mitochondria, highlighting the importance of restoring mtROS signalling for healthy ageing. For the sake of clarity, we only depicted CI-generated ROS in young mitochondria. Nevertheless, it is evident that CIII also plays a significant role in generating ROS in young and healthy individuals. In aged mitochondria, both CI and CIII are displayed to indicate the likelihood that multiple ROS generators (both specific and non-specific) are concurrently active.

Numerous examples in the literature demonstrate age-related changes in mitochondrial redox signalling, leading to severe disruptions in cellular homeostasis. In the fly brain and muscle, there is a significant increase in the levels of mtROS during ageing [28,61,113,126]. However, old and young brain mitochondria produce ROS in different ways. Young brain mitochondria have low levels of mtROS that are increased in response to specific types of stress. In contrast, old mitochondria continuously produce high levels of ROS and are less responsive to external stimuli [61,62]. In young flies, blocking the exit of electrons by knocking down subunits of CIV increases mtROS and causes interruptions in redox signalling. However, interventions targeting the entry of electrons (by knocking down CI subunits) prevent the generation of mtROS signals without increasing the amount of ROS [61,113]. A separate study reported an increase in mtROS in the flight muscle of Drosophila, which is crucial for extending lifespan resulting from CI depletion [112]. Along the same line, the knockout of the CIV assembly factor COX15 activates an ROS-dependent adaptation program in mouse muscle [124]. Interruption of this program by expressing AOX damages the antistress response and shortens survival. Similarly, in Drosophila flies, blocking mitochondrial redox signalling by expressing a mitochondrially targeted catalase damages the antistress response [30]. Conversely, boosting it by overexpressing SOD2 extends survival under stress and non-stress conditions [30,139]. Similarly, ectopic catalase expression in the mitochondria of astrocytes interrupts normal redox signalling, disrupting brain activity [29]. The former data indicate that young mitochondria produce low basal ROS levels and only increase ROS production in response to specific stimuli. Once the stress situation is over, mtROS levels return to normal. However, old mitochondria continually produce ROS and are unresponsive to stress (Figure 4). A recent paper showed that old fly mitochondria generate more ROS, triggering RET in CI [113]. Accordingly, restricting the inhibition of ROS-RET to flies older than 20 days using a novel, specific CI blocker (6-chloro-3-(2,4-dichloro-5-methoxyphenyl)-2-mecapto-7-methoxyquinazolin-4(3H)-one) leads to an extension of longevity. However, other manuscripts reported that ROS-RET extends fly lifespan [28], is instrumental for stress adaptation [30] and does not occur in the brain of flies older than 25 days [61]. Therefore, although different data support the loss of redox signalling during ageing, more studies are required to clarify how the former occurs mechanistically. An essential aspect of elucidating this mechanism is whether identical mechanisms generate ROS in both young and old mitochondria and whether there is a shift in the distribution of low- and highly reactive oxygen species in older individuals. Increased concentrations of hydroxyl radicals, peroxynitrite and other highly reactive free radicals may account for the accumulation of oxidative damage observed in aged individuals. Such an increase in the levels of aggressive ROS could be attributed to the liberation of iron and other metals from the catalytic centres of mitochondrial enzymes due to the amplified intensity of ROS signalling [140].

The proposed model presented in the previous figure (Figure 4) has not been experimentally verified, and there is indirect supporting and opposing evidence. For example, disruption of redox signalling explains why boosting antioxidants fails to positively impact lifespan in animal models [32] or human health in clinical trials [141]. Antioxidants neutralize oxidative damage but cannot restore mitochondrial redox signalling. The transcriptome of old flies presents many similarities with the transcriptome of flies exposed to hyperoxia [142], indicating an increase in the oxidation state of fly cells during ageing. This observation supports a model in which the interruption of redox signalling induces oxidative stress. However, the redoxome of neither flies nor mice consistently shows an increase in the number of oxidized cysteines in aged individuals. [77,143]. The former is surprising, considering the age-related decrease in reduced glutathione in insects and rodents [144,145]. On the one hand, the lack of consistent age-related modifications in the redoxome does not support a generalized interruption of redox signalling caused by mitochondrial dysfunction. On the other hand, specific tissues in mice exhibit age-related modifications in highly reactive cysteines in proteins associated with age-related diseases [143]. These specific modifications could account for the changes observed in the transcriptome of *Drosophila* without significant alterations in the redoxome. The consequences of losing redox signalling at the onset of ageing and age-related diseases deserve further investigation. Future studies must consider ROS as signals, not as simple metabolic byproducts. The majority of ROS-regulated processes discussed earlier have not been investigated in the context of ageing, a process during which mitochondrial function is notably impaired. This is not surprising, given that redox signalling remains a developing area of research, with limited studies dedicated to investigating its role in ageing, particularly in mammals. For example, an appropriate generation of mtROS is required to trigger sleep in fruit flies [146]. Accordingly, overexpression of catalase or AOX reduces sleep in transgenic flies, likely due to interruptions in redox signalling. However, it remains to be seen whether the age-related increase in mtROS generation is involved in disrupting sleep patterns in old animals [147]. To employ manipulations in redox signalling as a therapeutic strategy, it is crucial to first determine how the interruption of redox signalling affects ROS-regulated processes in both young and aged individuals. Second, it is essential to understand the underlying mechanisms driving changes in redox signalling and whether restoring redox signalling could potentially promote healthy ageing in humans.

## Data Availability

No original data was used in this review.

## Abbreviations

AOX	Alternative oxidase
CoQ	Coenzyme Q
CI	Complex I
CII	Complex II
CIII	Complex III
CIV	Complex IV
CV	Complex V
ETC	Electron transport chain
HIF1a	Hypoxia-inducible factor 1-alpha
IF1	ATPase inhibitory factor 1
IL-6	Interleukin 6
MAPK	Mitogen-activated protein kinase
MFRTA	The mitochondrial free radical theory of ageing
mROS	Mitochondrial reactive oxygen species
NDI1	NADH dehydrogenase internal 1
NOTCH	Neurogenic locus notch homolog protein
NOX	NADPH oxidase
OXPHOS	Oxidative phosphorylation
PRDX2	Peroxiredoxin-2
pmf	Proton motive force
RET	Reverse electron transport
S1QELs	Suppressors of superoxide production in CI
S3QELs	Suppressors of superoxide production in CIII
SOD1	Superoxide dismutase-1
SOD2	Superoxide dismutase-2
TGF-beta	Transforming growth factor beta
TNF	Tumour necrosis factor
